# Diagnostic value of the wnt target and cancer-associated blood biomarker hPG80: ONCOPRO case-control prospective study

**DOI:** 10.1186/s40364-025-00793-z

**Published:** 2025-07-01

**Authors:** Benoit You, Sebastien Couraud, Philippe Ceruse, Lionel Badet, Philippe Paparel, Alice Durand, Marielle Guillet, Philippe Merle, Gaelle Lescuyer, Charles-André Philip, Francois Ducray, Mathieu Pioche, Lionel Karlin, Jean-Christophe Lifante, Olivier Glehen, Pierre-Adrien Bolze, Marion Chauvenet, Carole Langlois-Jacques, Fabien Subtil, Marie Piecyk, Aurore Carrot, Dominique Joubert, Alexandre Prieur, Berengere Vire, Sara Calattini, Lea Payen

**Affiliations:** 1 Lyon University Hospital, IC-HCL, Lyon University, Lyon, France; 2 Medical Oncology, IC-HCL, EPSILYON, Université Claude Bernard, CICLY, Lyon 1, Univ Lyon 1, Lyon, France; 3 Pneumology, Lyon-Sud Hospital, URCOT, CICLY, Lyon, France; 4 Head and Neck Surgery, Croix Rousse Hospital, CICLY, Lyon, France; 5 Urology, Edouard Herriot Hospital, Lyon, France; 6 Hépatogastroentérologie et Endoscopie Digestive, E Herriot Hospital, Lyon, France; 7 Hepatology and Gastroenterology, Croix-Rousse Hospital, Lyon, France; 8 Center for Innovation in Cancerology of Lyon (CICLY) ER 3738, Faculty of Medicine and Maieutic Lyon Sud, Claude Bernard University Lyon I (UCBL1), Institut of Pharmaceutical and Biological Sciences of Lyon (ISPB), Oullins, 69921 France; 9 Gynecology and Obstetrics, Hopital Femme Mere Enfant, Lyon, France; 10 Neurology, East Hospital, Lyon, France; 11 Hematology, Lyon-Sud Hospital, Lyon, France; 12 Endocrine Surgery, RESHAPE, Lyon, France; 13 Visceral and Digestive Surgery, Lyon-Sud Hospital, CICLY, Lyon, France; 14 Hepatology and Gastroenterology, Lyon-Sud Hospital, Lyon, France; 15 Service de Biostatistique, Pole Santé Publique, Lyon, France; 16 Université Claude Bernard Lyon 1, LBBE, UMR 5558, CNRS, VAS, Villeurbanne, France; 17 Progastrin Manufacturing, Cap Sigma, 1642 rue de la Valsière, Grabels, 34790 France; 18 LBMMS, Université Claude Bernard Lyon 1, Univ Lyon 1, CICLY, Lyon, 69003 France; 19 Gynecology and Obstetrics, Lyon-Sud Hospital, CICLY, Lyon, France

**Keywords:** Biomarkers, tumor, Early detection of Cancer, Wnt signaling pathway, Prospective studies

## Abstract

**Background:**

hPG_80_ (circulating progastrin), initially recognized for its oncogenic properties due to its direct link to the Wnt signaling pathway, is secreted by cancer cells and detectable in the blood of cancer patients. The ONCOPRO centralized case-control study (NCT03787056) was designed to prospectively evaluate the diagnostic utility of hPG_80_ in patients with 16 different types of cancer.

**Methods:**

hPG_80_ levels were measured in 421 patients with 16 newly diagnosed cancers (median age 65.6 years old) using the DxPG80.Lab kit (Biodena Care). The diagnostic performance of hPG_80_ (the primary endpoint) was assessed by comparing baseline hPG_80_ levels in cancer patients with those of 330 asymptomatic aged-matched healthy subjects from the general population.

**Results:**

Between 2018 and 2022, a total of 506 cancer patients were enrolled in the study, with 421 assessable across 16 distinct cancer cohorts. hPG_80_ concentrations were significantly higher in cancer patients compared to the healthy population (median 3.8 [IQR: 1.0-11.1] vs. 1.9 [IQR: 0.6–4.2] pM, *P* < 0.0001). hPG80 levels were not impacted by renal failure, liver dysfunction, or cancer-associated inflammation. The diagnostic accuracy in cancer patients was ROC AUC 0.63, 95% CI = [0.59–0.67]. The highest diagnostic accuracy was seen in patients with lung cancer (AUC 0.75, 95% CI [0.68–0.82]; specificity = 88% for hPG_80_ > 7.73 pM in patients aged > 58 years old) and hepatocellular carcinoma HCC (ROC AUC 0.75, 95% CI [0.66-083]; specificity = 88% for hPG_80_ > 7.73 pM in patients aged > 58 years old).

**Conclusions:**

This large prospective study confirms that cancer patients have significantly higher hPG_80_ blood concentration compared to the healthy population. Incorporating this straightforward ELISA assay into screening programs is warranted.

**Trial registration:**

NCT03787056.

**Supplementary Information:**

The online version contains supplementary material available at 10.1186/s40364-025-00793-z.

## Introduction

According to a study done by the World Health Organization in 2021, cancer is one of the top two leading causes of death globally [1]. Among various strategies aiming at improving cancer prognosis, one promising approach involves the development of screening programs capable of detecting cancer at an early stage when the disease is still curable [2]. 

Recently, several multi-cancer blood assays have emerged, attracting significant interest from the scientific and medical communities. These assays hold potential for integration into screening programs to identify patients with cancers originating from different tissues. Most of these assays are based on circulating tumor DNA (ctDNA) technology, which, despite its promise, faces limitations regarding complexity, cost, and sensitivity for routine clinical use [3]. 

hPG_80_ (extracellular circulating progastrin) is emerging as a promising blood-based biomarker for various cancers, with significantly higher levels observed in cancer patients compared to healthy asymptomatic individuals [4]. In healthy individuals, progastrin is produced by G cells of the stomach, and is the precursor of gastrin. In tumor cells, the GAST gene, which encodes hPG_80_, is directly regulated by the WNT/β-catenin oncogenic pathway, a pathway activated in many cancers [5]. Unlike in stomach G cells, tumor cells produce progastrin but do not process it into mature gastrin. This secreted unprocessed form, named hPG_80_, is easily measurable using a cost-effective and straightforward ELISA method [4]. Several studies showed the strong relationships between GAST gene activation, WNT pathway activation, and hPG80 expression in adenoma and cancer cells [6–8]. 

hPG_80_ plays a pivotal role in tumorigenesis by regulating cancer stem cell functions [9, 10], promoting angiogenesis [11], and influencing apoptosis [12]. Retrospective studies have established correlations between elevated hPG_80_ levels and poor survival outcomes in metastatic renal cell carcinoma (mRCC) [13], hepatocellular carcinoma (HCC) [14], breast cancer [10], and glioblastoma [15]. 

However, these findings were derived from retrospective analyses. A prospective study was necessary to validate these results, identify cancer types with the highest diagnostic accuracy for hPG_80_, and strengthen its potential for clinical application.

The ONCOPRO study (NCT03787056) was a prospective case-control study designed to assess: *(1)* the diagnostic value of hPG_80_ by comparing blood concentrations of this marker in patients with 16 newly diagnosed cancer types to those in asymptomatic healthy subjects (primary endpoint); and *(2)* the monitoring potential of hPG_80_ by evaluating its longitudinal kinetics during treatment (secondary endpoints).

The results for the primary endpoint are presented here.

## Patients and methods

### Cancer patient case cohorts

ONCOPRO study (NCT03787056) has been conducted in 17 medical or surgical cancer departments of a single institution with a centralized coordination (Lyon University Hospital, HCL, Lyon, France). The patients were enrolled in 16 cohorts, including 6 large cohorts with minimum 50 patients (Non-Small Cell lung Carcinoma (NSCLC) & Small Cell Lung Carcinoma (SCLC); Breast; Renal; Prostate; Hepatocellular carcinoma (HCC); and Head-and-Neck carcinomas) and 10 small explorative cohorts including 10 patients (Colorectal; Thyroid; Pancreas; Glioblastoma; Endometrial; Bladder; Superficial esophageal-gastric; Lymphoma; and Gastric carcinomas).

The selection of targeted cancers was based on the available data about hPG_80_, and clinician perception about the unmet medical need for early diagnosis tests. Large cohorts were meant to assess the diagnostic value in these selected cancers with sufficient statistical power, whilst the small cohorts were more explorative.

**Fig. 1:**
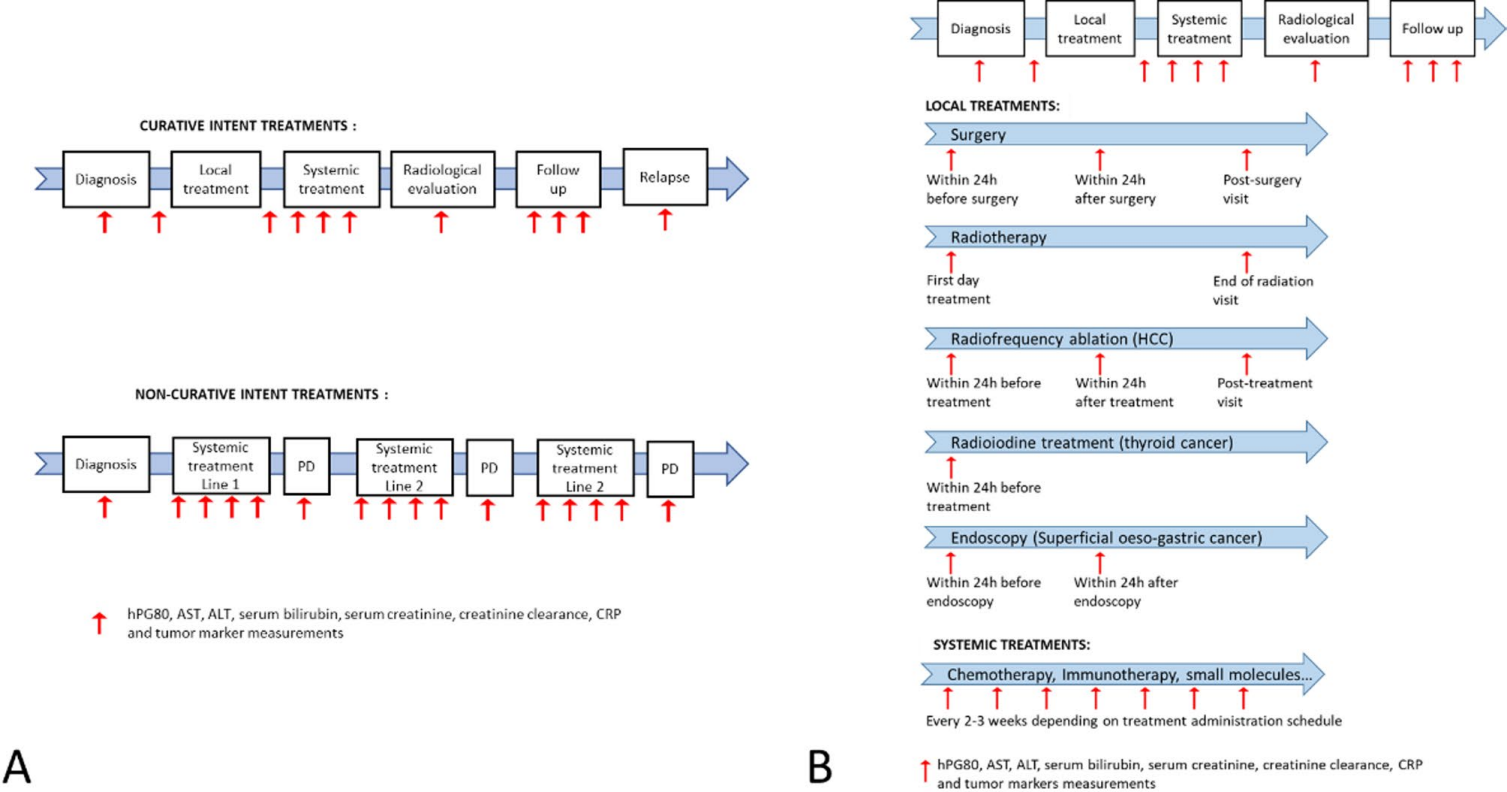
ONCOPRO study design with biological sampling strategies for (**A**) curative intent or non-curative intent treatments, and (**B**) during systemic or local treatments

To be enrolled and considered assessable, the patients had to be older than 18 years, diagnosed with a histologically and/or cytologically documented cancer, planned to be treated with a curative or non-curative intent strategy. Patients enrolled in “curative intent strategy” cohorts had to be naïve of anticancer treatment, whilst the patients enrolled in “non-curative intent strategy” cohorts had to be naïve of treatment in the non-curative setting (but could have been previously treated for cancer in curative setting) (Fig. 1).

The other inclusion criteria included measured creatinine clearance > 30 mL/min or creatinine ≤ 1.5 x ULN; aspartate aminotransferase (AST) and alanine aminotransferase (ALT) ≤ 2.5 x ULN (or ≤ 5 x ULN, in case of liver metastases); and serum bilirubin ≤ 1.5 x ULN. Specific biological criteria applied for the hepatocellular carcinoma cohort (Suppl Material).

Exclusion criteria for curative-intent cohorts comprised a history of previous cancers, except for adequately treated non-melanoma skin cancer, curatively treated in-situ cancer of the cervix with no evidence of disease for ≥ 5 years. The same criteria applied for non-curative-intent groups, except that patients with a recurrent cancer could have previously been treated with a curative intent treatment.

In addition, some patients were proposed to also participate in one of the two kinetic cohorts designed to better understand the longitudinal kinetics of hPG_80_ in short time windows: *(1)* nycthemeral cohort meant to assess the hPG_80_ changes during a 24 h time period with respect to meals; *(2)* a post-operative cohort, proposed to patients treated with initial curative surgery and meant to assess the early dynamics of hPG_80_ in the 24 h following the surgical procedure.

All patients gave their consent and signed the informed consent. The protocol, the written information sheet and the consent form for the study obtained a favorable opinion from to ethics committee Ile de France VI on October 3rd 2018. This notification was transmitted to the French health authority ANSM. All Protocol amendments were approved by the same ethic committee.

### hPG_80_ sampling times for cancer cohorts

Circulating hPG_80_ sampling strategy was individually adjusted to the patient treatment schedule (Fig. 1). The general principle was that hPG_80_ was sampled at baseline before any treatment (for the primary objective), and then at each hospital visit for those treated with intra-veinous systemic treatment, before and after surgery in the case of curative surgery, before and after radiation therapy when applied, at each consultation visit for oral treatment, and during the follow-up period. For curative-intent cohort patients, hPG_80_ was monitored during treatment, follow-up, and at relapse if any, for a maximum of 5 years. For non-curative-intent cohort patients, hPG_80_ was monitored on 3 consecutive treatment lines (Fig. 1).

In addition to hPG_80_, the following biomarkers were measured at the same times: AST; ALT; serum bilirubin (in addition to albumin, factor V et prothrombin time for HCC), serum creatinine, creatinine clearance, C-Reactive Protein (CRP); serum tumor markers for specific cohorts (CA15-3 and Carcinoembryonic antigen (CEA) for breast cancer, Prostate-specific antigen (PSA) for prostatic cancer, alpha foetoprotein (AFP) for HCC, CA19-9 and CEA for colorectal, gastric, superficial oesophago-gastric and pancreatic cancers, thyroglobulin for thyroid cancer, and CA125 for ovarian cancer and endometrial cancers).

In the nychthemeral cohort, hPG_80_ was sampled before start of treatment on a 24-hour period, starting on day 1 at 11 am, 12 pm, 2 pm, 5 pm, 6 pm, 9 pm, 9 to 10 pm, and on day 2 at 7 am and 9 am, to assess a potential impact of meal intakes on hPG_80_ concentrations (Suppl Fig. 1). In the post-operative kinetic cohort, hPG_80_ was sampled just at the end of surgery, + 30 min, + 60 min, + 2 h, + 4 h, + 6 h, + 12 to 24 h (Suppl Fig. 1).

### Asymptomatic healthy subjects control group

In a parallel prospective study, asymptomatic healthy subjects with no history of cancer were recruited to provide data on hPG_80_ blood levels, constituting the control group. Their characteristics were matched to those of recruited cancer patients for age and gender. The exclusion criteria included: any known personal history of cancers, or pre-cancerous lesions such as atypical dysplasia, in situ carcinomas; active smoking or previous smoking habit > 5 Pack-Year); a medical daily treatment (except for contraceptive, endocrine treatment for menopause, anti-hypertensive treatment, psychotropic treatment and anti-lipemic treatment provided that the inherent disease had been under control for at least 3 months). All patients gave their consent and signed the informed consent. Ethical approval was granted by the French health authority ANSM and the ethics committee CPP (ID-RCB N°0.2021-A02356-35). All Protocol amendments were approved by the same authorities and explained in the protocol.

### Centralized hPG_80_ level measurements in the blood samples

hPG_80_ blood levels were centrally measured in the multisite laboratory of medical biology and pathology, in the “Cancer Pathology Medical Unit” of Biochemistry and Molecular Biology department of Lyon University Hospital. The ELISA DxPG_80_.Lab kit (Biodena Care, Grabels, France) was used to measure hPG_80_ levels in all blood samples according to the manufacturer instructions described previously [16]. Briefly, the limit of Detection (LoD) was set at a hPG_80_ concentration of 1.2 pM and the limit of Quantitation (LoQ) at a hPG_80_ concentration of 3.3 pM. The inter- and intra-assay coefficients of variation (CV%) were below 10%. No cross-reactivity was detected with gastrin-17, Gastrin-Gly or CTFP (C-Terminus Flanking Peptide). No cross-reactivity was detected with other blood biomarkers such as CA125, CEA or PSA. No interference was detected with chemicals and endogenous compounds such as SN-38, 5-FU or triglycerides, cholesterol or hemoglobin.

### Objectives and statistical analyses

The primary objective was to characterize the cancer diagnostic accuracy of hPG_80_ assay at baseline, before any treatment, in cancer patients with respect to control cases, using Receiver Operating Characteristics (ROC) curves. Considering 421 enrolled cancer patients (pooled cancer cohorts) and 330 asymptomatic healthy subjects, the expected precision for a 95% confidence interval of the area under the ROC curve of the hPG_80_ was 0.022 for an expected AUC of 0.9. Calculations were performed assuming that plasma biomarker value distribution would follow an exponential distribution in the diseased and non-diseased subjects [17]. Under the same assumptions (AUC = 0.9, exponential distribution of the hPG_80_), the large cohort of 50 patients will enable to obtain a precision of 0.06 for the 95% confidence interval of this area.

The secondary objectives were *(1)* to assess the influence of patient covariates (renal function, liver function, age, CRP…) on hPG_80_ values before any treatment as well as during multi-modal treatments, during follow up and at relapse; *(2)* to explore the variability of hPG_80_ after meals (nycthemeral cohort), and after surgery; *(3)* to characterize the diagnostic accuracy by cancer origin within the large cohorts, and explore the hPG_80_ concentration associated with a 90% specificity; *(4)* to estimate the prognostic value of hPG_80_ modeled kinetics for recurrence free, progression free and overall survivals; and *(5)* to characterize the kinetics of hPG_80_ during multi-modal treatments (chemotherapy, surgery, radiotherapy, other…), during follow-up and at relapse, in localized or metastatic cancers.

Five main amendments were made between June 2018 and January 2023. Two of them introduced significant changes in the composition of cohorts to optimize the recruitment rate (e.g. suppression of a melanoma cohort replaced by a NSCLC cohort treated with immunotherapy; increase of the head-and-neck cancer cohort up to 50 patients; suppression of the “weekly cohort” meant to assess the hPG_80_ kinetics on a weekly basis during the first month of treatment) (Suppl Material).

As a consequence, the number of patients to enroll increased up to 420 (compared to the 410 initially planned).

In the present manuscript, we present the primary objective, and some secondary objectives related to the baseline hPG_80_ values.

Differences in hPG_80_ levels between cases and controls were evaluated using the Wilcoxon rank-sum test. The diagnosis discriminative accuracy of hPG_80_ levels in patients with cancer compared to asymptomatic healthy subjects was quantified by the Area under the ROC curve (AUC) with its 95% confidence interval. The correlation between hPG_80_ and baseline characteristics was quantified by the Spearman correlation coefficient. Analyses were performed using SAS version 9.4 and R version 4.1.2. The level of significance was set at *P* < 0.05.

## Results

### Clinical characteristics of the enrolled cancer patient cases and asymptomatic healthy subjects

**Fig. 2:**
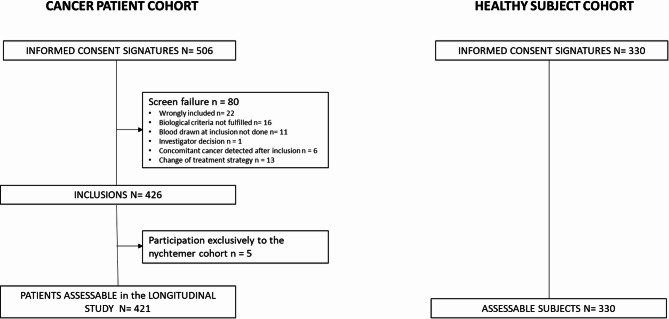
Flowchart of ONCOPRO study

Between December 2018 and June 2022, 421 patients out of 506 patients enrolled were assessable as per study criteria (Fig. 2). The main characteristics of assessable patients are presented in Table 1. The median age was 65.6 [IQR: 58.2–72.9], and the gender distribution was 59.6% men/ 40.4% women.

**Table 1:** Characteristics of cancer patient cases

	*N*	Age			Gender males (%)
		Median	Q1	Q3	
TOTAL	421				251 (59.6)
Breast cancer	50	54.3	46.1	67.1	0 (0%)
* Curative intent*	*30*	*49.5*	*41.2*	*56.6*	*0*
* Non-curative intent*	*20*	*67*	*54.4*	*76.7*	*0*
Renal cancer	50	61.6	55.5	71.2	33(66%)
* Curative intent*	*25*	*62.6*	*54.1*	*69.8*	*17*
* Non-curative intent*	*25*	*60.4*	*58*	*72.3*	*16*
Prostate cancer	50	69.6	64.8	74.6	50(100%)
* Curative intent*	*25*	*66.1*	*64.6*	*69.7*	*25*
* Non-curative intent*	*25*	*73.3*	*65.5*	*77.8*	*25*
Lung cancer	70	66.5	60.5	71.7	42(60%)
* Curative intent*	20				10 (50%)
* Stage I*	5	*63.3*	*63.1*	*68.4*	2
* Stage II*	*1*	*66.3*	*66.3*	*66.3*	1
* Stage III*	*7*	*67.3*	*65.3*	*72.6*	4
* Unknown*	7	*72.8*	*67.4*	*74.1*	3
* Non-curative intent*	50				32 (64%)
* Stage III*	*1*	*66.3*	*66.3*	*66.3*	*1*
* Stage IV*	*48*	*67.3*	*65.3*	*72.6*	*30*
* Unknown*	*1*	*69.9*	*69.9*	*69.9*	*1*
Hepatocellular Carcinoma	50	71.4	64.5	75	39(78%)
* Curative intent*	*40*	*71.9*	*65*	*75.5*	*31*
* BCLC score 0*		*60.9*	*59.7*	*62.1*	*2*
* BCLC score A*		*72.3*	*65.5*	*748*	*20*
* BCLC score B*		*75*	*69.1*	*767*	*9*
* Non-curative intent*	*10*	*66.9*	*62.9*	*71.9*	*8*
* BCLC score B*		*64.1*	*62.6*	*69.7*	*7*
* BCLC score C*		*71.9*	*71.9*	*71.9*	*1*
Head and Neck cancer	50	63.0	56.9	70.2	36(72%)
* Curative intent*	*30*	*62*	*56.3*	*70.2*	*19*
* Non-curative intent*	*20*	*63.2*	*58.4*	*69.9*	*17*
Gastric cancer	10	75.3	64.9	79.6	7(70%)
Colorectal cancer	10	61.3	56.9	70.7	4(40%)
Pancreatic Cancer	10	67.7	56	74.5	6(60%)
Ovarian Cancer	10	66.6	46.9	69.2	0(0%)
Glioblastoma	10	64.4	55.6	72.2	7(70%)
Endometrial cancer	10	69.7	65.6	73.3	0(0%)
Bladder Cancer	10	69.7	59.5	73	5(50%)
Superficial oeso-gastric cancer	11	73.3	63.9	81.4	11(100%)
Diffuse large B-cell lymphoma	10	67.5	64	72.2	10(100%)
Thyroid cancer	10	34.3	32.5	45.3	1(10%)

Barcelona-Clinic Liver Cancer (BCLC)

Between January and April 2022, 330 asymptomatic healthy subjects were enrolled in parallel, with matched characteristics to cancer cases for age and gender distribution.

**Fig. 3:**
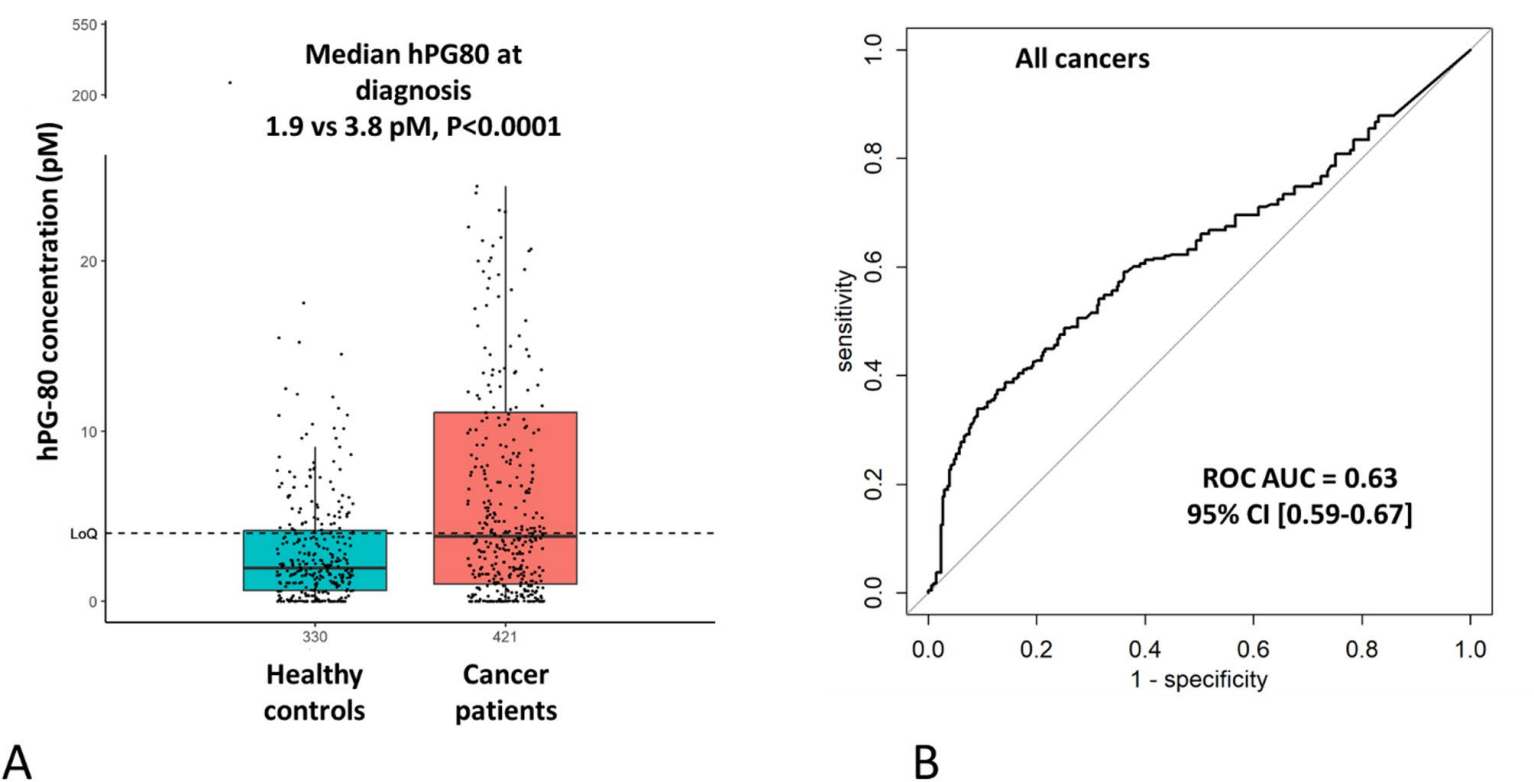
Diagnostic value of hPG_80_ in cancer patient cases compared to asymptomatic healthy subjects. (**A**) Box-plots of hPG_80_ concentration. (**B**) ROC curve area under the curve

Compared to asymptomatic healthy subjects, the cancer patients had statistically higher baseline value of hPG_80_ when all cohorts were pooled together (median, 3.8 [IQR: 1.0-11.1] pM vs. 1.9 [IQR: 0.6–4.2] pM, *P* < 0.0001) (Fig. 3A). The area under the ROC curve was 0.63 (95% CI = [0.59–0.67]), and was statistically different from 0.5 (Fig. 3B).

**Fig. 4:**
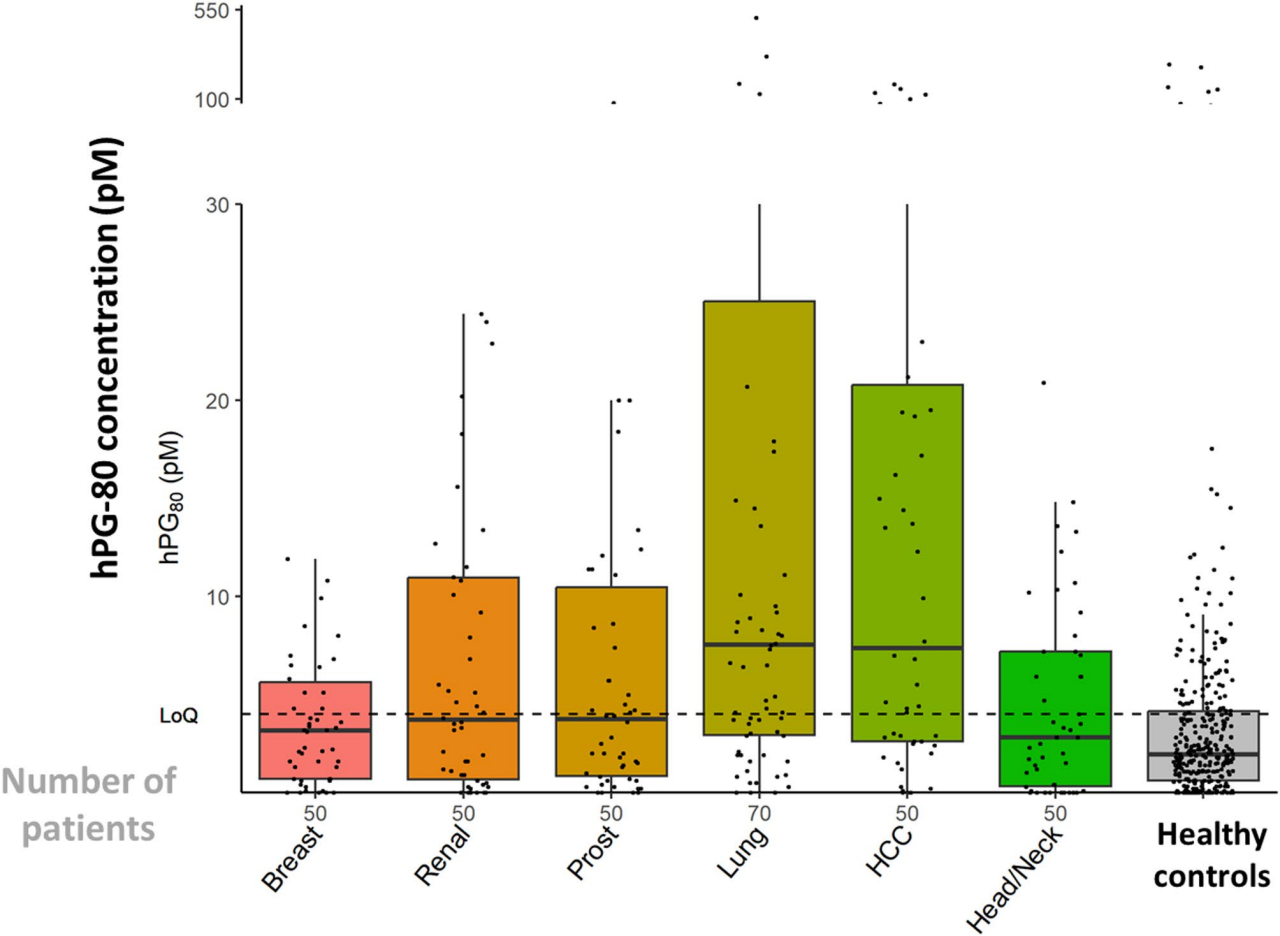
hPG_80_ concentrations in the large cancer patient cohorts and the asymptomatic healthy subjects. The number of patients is indicated on the x-axis

hPG_80_ concentrations were not homogenously elevated in all the cohorts (Suppl Fig. 2, and Suppl Fig. 3). As shown on the Fig. 4, displaying the baseline values of hPG_80_ in the large cohorts, the patients with lung cancers and with HCC had the highest baseline titers.

There was a significant and weak correlation between increasing patient age and higher hPG_80_ concentrations in both the cancer patient cases (*R* = 0.24, *P* < 0.001) (Suppl Fig. 4) and asymptomatic healthy subjects (*R* = 0.22, *P* < 0.001) (Suppl Fig. 5). Men tended to have higher hPG_80_ concentrations compared to women, but the difference was not statistically significant (median, 4.2 [IQR: 1.1–13.4] vs. 3.4 [IQR: 0.7–9.9] pM, *P* = 0.087) (Suppl Fig. 6).

No clinically relevant correlation was found between hPG_80_ concentrations and liver function (ALT, *R* = 0.03, *P* = 0.51; AST, *R* = 0.11, *P* = 0.029; bilirubin, *R* = -0.02, *P* = 0.67), renal function based on serum creatinine levels (*R* = 0.23, *P* < 0.001), or inflammatory status assessed by CRP (*R* = 0.11, *P* = 0.14) (Suppl Figs. 7 to 9).

In the nychthemeral cohort, 15 patients were enrolled, including 5 patients with breast cancers, 4 with renal cancers, 4 with bladder cancers, 1 with ovarian cancer, and 1 with endometrial cancer. hPG_80_ concentration was not impacted by meal intakes (Suppl Fig. 10). In the post-operative cohort, 12 patients were enrolled, exclusively head-and-neck cancers. Out of 6 patients with elevated pre-operative hPG_80_ > LoQ, a decline to concentration < LoQ was observed in 5 patients 2 h post-surgery (83%) (Suppl Fig. 11).

### Diagnostic value of hPG_80_ in the large cohorts of interest

Among the 6 large cohorts, the diagnostic accuracy of hPG_80_ was particularly more promising in patients with lung cancers and HCC aged > 1st quartile (58 years on the cancer population), an age at which cancer risk increases.

**Fig. 5:**
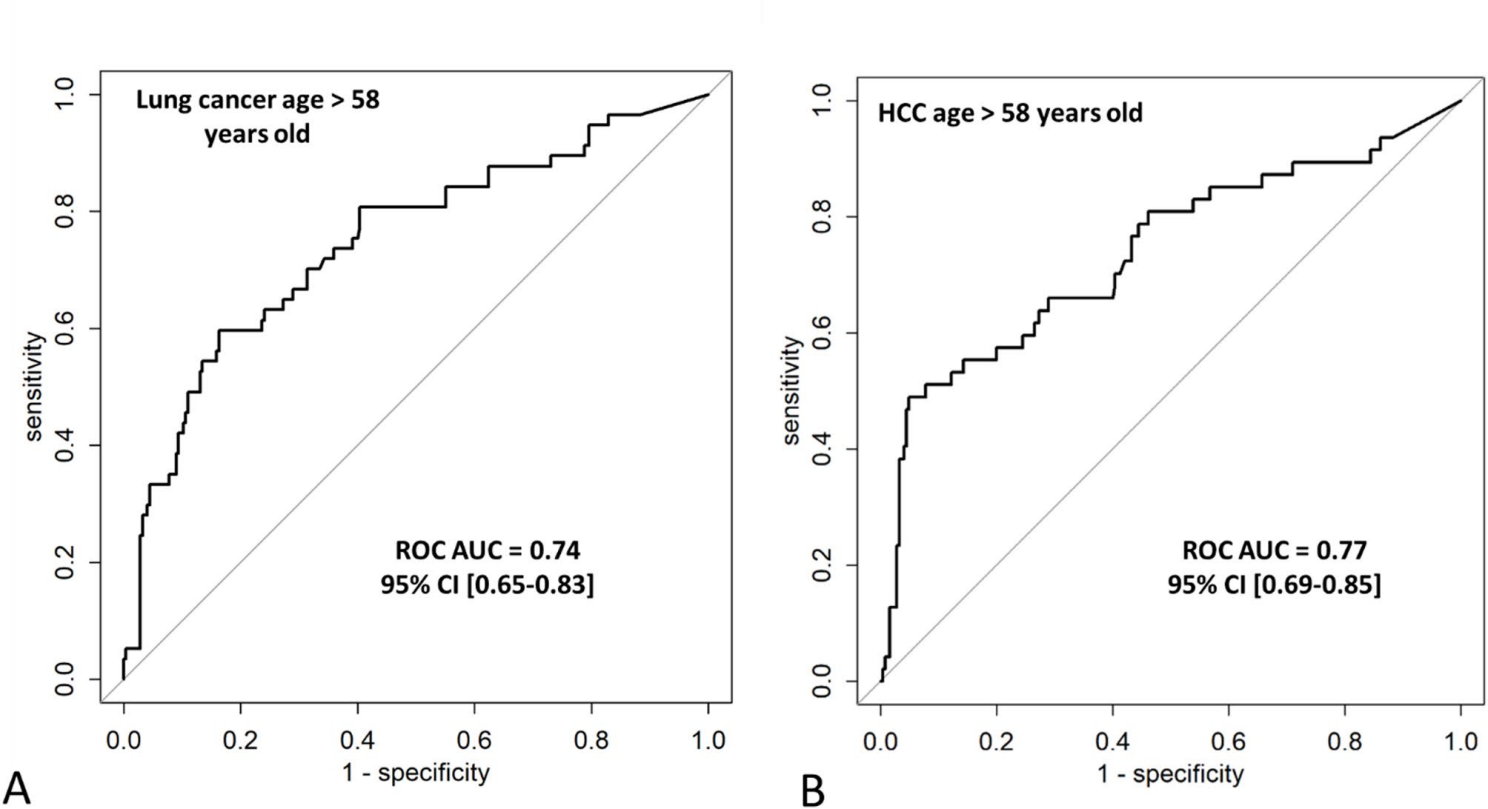
Diagnostic value of hPG_80_ in patients with lung cancer (**A**) and HCC (**B**) aged 58 years or older, compared to asymptomatic healthy subjects

Indeed, in 56 patients with NSCLC or SCLC cancers aged > 58 years, the ROC AUC was 0.75, 95% CI = [0.67–0.82] with a 49% sensitivity and 88% specificity for hPG_80_ > 7.73 pM (Fig. 5A). hPG_80_ was found elevated in the 5 patients with early stage I disease (median, 26.5 pM [IQR: 8.1–28.5]). In the NSCLC cancer patients from the non-curative cohort where the data were available, the baseline value of hPG_80_ was not significantly different according to the smoking status (active, *n* = 20; former, *n* = 22; never, *n* = 5, *P* = 0.83), or the molecular status regarding *TP53*, *K-RAS*, or *B-RAF* mutations in the NSCLC cancer patients on tumor tissue or circulating tumor DNA (Suppl Tables 1, and Suppl Figs. 12 and 13).

Similarly, in the 47 patients with HCC aged > 58 years, the ROC AUC was 0.74, 95% CI = [0.65–0.83], with a 51% sensitivity and 88% specificity for hPG_80_ > 7.73 pM (Fig. 5B). The diagnostic value of AFP was overlapping compared to hPG_80_, (ROC AUC, 0.77, 95% CI = [0.69–0.85]) (Suppl Fig. 14). Among the 39 patients with AFP-negative HCC at diagnosis (< 20 ng/mL), 51.2% patients had hPG_80_ > 7.73 pM, meaning that hPG_80_ could potentially compensate the false-negative AFP. The baseline value of hPG_80_ was not different according to the Barcelona-Clinic Liver Cancer (BCLC) score (0, *n* = 2; A, *n* = 28; B, *n* = 19; C, *n* = 1; *P* = 0.59) (Suppl Fig. 15), or alcohol consumption (No, *n* = 38; Yes, *n* = 12; *P* = 0.68).

In other large cohorts where serum biomarkers are routinely used—such as PSA for prostate cancer and CA 15 − 3 or CEA for breast cancer—the added diagnostic value of hPG_80_ was also evaluated. In prostate cancer, the diagnostic performance of PSA alone was already high (ROC AUC: 0.97; 95% CI: 0.94–0.99), and the addition of hPG_80_ did not further improve the overall diagnostic accuracy (combined ROC AUC: 0.97; 95% CI: 0.94–0.99).

Similarly, in breast cancer, the diagnostic values of both CA 15 − 3 and CEA were limited (ROC AUC: 0.65; 95% CI: 0.55–0.75 and 0.61; 95% CI: 0.52–0.72, respectively), and the addition of hPG_80_ provided no improvement (CA 15 − 3 + hPG_80_: ROC AUC 0.67; 95% CI: 0.57–0.76; CEA + hPG_80_: ROC AUC 0.61; 95% CI: 0.52–0.72).

## Discussion

The development of multi-cancer early detection (MCED) tests capable of screening for multiple cancers from a simple blood sample has become a major area of interest within the scientific community [18, 19]. Most of these assays are based on circulating tumor DNA. Their clinical validity and utility have been assessed in large prospective clinical trials. The DETECT-A study enrolled approximately 10,000 women without a known cancer diagnosis to evaluate a panel of 16 genes using circulating cell-free DNA (cfDNA), along with 8 circulating proteins, followed by confirmation through positron emission tomography (PET)–computed tomography (CT) scanning. The results demonstrated a sensitivity of 15.6% with a specificity of 99.6% [20]. Similarly, the PATHFINDER study assessed a targeted methylation sequencing assay using cfDNA, detecting cancer signals in 1.4% (92/6621) of prospectively recruited participants, with a specificity of 99.1% and a positive predictive value (PPV) of 38.0% [21]. Other prospective clinical trials are currently underway to evaluate not only the benefit-to-harm ratio, but also the cost-effectiveness of these expensive MCED technologies which is still unclear [22–25]. 

In this context, hPG_80_ has been developed as a novel blood-based multi-cancer diagnostic biomarker, offering significant advantages over existing assays due to its simpler implementation. The hPG_80_ assay, based on an ELISA test, can be performed in any standard medical laboratory, making it a far more cost-effective option [16]. Following the highly promising results from several retrospective studies demonstrating the diagnostic potential of hPG_80_ across multiple cancer types, evaluating its clinical utility through a prospective case-control study was warranted [4, 10]. 

Conducting a large-scale prospective study involving nearly 500 patients across 16 different cancer types and 17 cancer departments within a single institution was a significant organizational achievement. Centralized coordination and standardized hPG_80_ measurements were implemented to ensure uniformity in both data collection and procedural execution, overcoming the logistical challenges of such a complex study design. The results indicate that hPG_80_ levels were significantly higher in cancer patients compared to asymptomatic healthy subjects, and this difference was not attributable to altered renal or liver function, nor to inflammatory status. We conclude that the elevated hPG_80_ levels are likely attributable to the presence of cancer. The post-surgery decrease of hPG_80_ observed in 83% of patients with initial high levels would support this assumption, as previously reported in glioblastoma and peritoneal carcinomatosis [4, 15]. Although the diagnostic discriminative accuracy was statistically different from 0.5, it was lower than anticipated based on retrospective studies when all cancer cases were evaluated [4, 10]. 

In this study, the most clinically relevant findings were observed in patients over 58 years of age (age > first quartile in our cohort) with lung cancer and hepatocellular carcinoma (HCC). Indeed, the diagnostic accuracy was high for lung cancer patients, with a ROC curve AUC of 0.75. This high diagnostic accuracy of hPG_80_ may be hypothetically explained by the robust activation of the WNT signaling pathway, which typically occurs through the overexpression of WNT ligands (e.g., Wnt1, Wnt3a) or their receptors (e.g., Frizzled-7), leading to downstream transcriptional activation of WNT target genes, including GAST [26]. In our study, at a cutoff value of 7.73 pM, the specificity was 90%, with a sensitivity of 49%. As a consequence, hPG_80_ could be a valuable addition to the current screening strategies, complementing low-dose CT scans. By improving specificity in patients with suspicious lung lesions, hPG_80_ may be an interesting biomarker for non-invasive diagnosis of lung nodules [27]. 

For hepatocellular carcinoma (HCC), the diagnostic accuracy was similarly high, with an ROC curve AUC of 0.74. This observation is consistent with the literature, as HCC is frequently characterized by somatic mutations in CTNNB1, the gene encoding β-catenin. These mutations result in constitutive activation of β-catenin and subsequent uncontrolled transcription of downstream WNT target genes [28]. At a cut-off of 7.73 pM, the specificity was 90%, with a sensitivity of 48%. Moreover, over 50% of patients with AFP-negative HCC at diagnosis (AFP < 20 ng/mL) exhibited elevated hPG_80_ concentrations. This finding aligns with previous reports indicating that AFP is non-informative in 50–70% of HCC cases, and that hPG_80_ is independently elevated in more than 60% of these patients [14, 29]. These results support the potential role of hPG_80_ as a complementary biomarker to AFP for improving the diagnostic accuracy in HCC.

We believe that hPG_80_ could be a valuable complement to AFP measurement in patients suspected of having HCC [29]. 

While the diagnostic value of hPG_80_ shows promise for lung cancer and HCC in patients aged 58 years or older, the study was limited by a small sample size and lower diagnostic accuracy in patients with other cancer types. These findings suggest that the spectrum of cancers detectable by the hPG_80_ assay is likely narrower than initially anticipated when it was developed as a multi-cancer early detection biomarker. Similar to other MCED tests currently in development, the accuracy of cancer detection varies depending on the type of cancer [19, 23]. 

These outcomes will help guide the further development of hPG_80_ as a biomarker. The exclusion of patients with lung cancer and HCC younger than the first quartile age (58 years) is open to criticism but is justified, as the cancer risk in younger patients is obviously lower and less relevant from a macroeconomic perspective. Some discrepancies were observed between our results and those of retrospective studies for certain cancers. For example, hPG_80_ levels were low in the exploratory glioblastoma cohort (median, 0.3 pM (IQR 0.00-1.85), *n* = 10) compared to the retrospective study published by Doucet et al. where the median hPG_80_ concentration was 5.37 pM (IQR 0.00-13.90). These discrepancies underscore the need for additional data, which is currently being collected from ongoing prospective studies. Indeed, blood hPG_80_ concentrations are being measured in patients with high-grade glial tumors (PROGLIO, NCT05157594), and those with neuroendocrine tumors (PRO-NEM1, NCT06430021; and NCT05724108). The high variability of hPG_80_ suggests that this biomarker should likely be developed as a complementary test rather than as a standalone tool, to overcome the limited sensitivity and specificity of existing screening methods. The prognostic value of baseline hPG_80_ regarding progression-free survival and overall survival— a secondary endpoint in the ONCOPRO study—could not be assessed due to the insufficient number of events. This will be addressed in future reports. Additionally, the present study did not evaluate the monitoring value of hPG_80_, which is a key secondary endpoint of ONCOPRO. This will be assessed on a cohort-by-cohort basis. Finally, other multi-cancer early detection biomarkers, such as ctDNA, nucleosomes, and epigenetic circulating markers will be assessed on the ONCOPRO samples to investigate their diagnostic and monitoring potential, as well as their possible complementarity with hPG_80_.

In conclusion, the ONCOPRO case/control prospective study was successfully conducted across 17 departments within a single institution, with centralized coordination. hPG_80_ concentrations were effectively measured to assess its diagnostic value in patients with 16 different types of cancer. The study confirmed that hPG_80_ levels are significantly higher in cancer patients compared to asymptomatic healthy subjects, independent of renal or liver function and inflammatory status. Despite some variability, the diagnostic accuracy of hPG_80_ was statistically significant. The most clinically relevant findings were observed in patients with lung cancer and HCC, where high ROC AUC values and high specificity suggest that hPG_80_ could serve as a complementary biomarker to existing screening tools in these cancers.

## Electronic supplementary material

Below is the link to the electronic supplementary material.

Supplementary Material 1

## Data Availability

No datasets were generated or analysed during the current study.
