# Supplementary material for: Diagnostic value of the wnt target and cancer-associated blood biomarker hPG80: ONCOPRO case-control prospective study
Source: Biomark Res. 2025 Jul 1;13:91. doi: 10.1186/s40364-025-00793-z (PMC12218953; doi:10.1186/s40364-025-00793-z)
Supplement: Supplementary file 1 — Supplementary Material 1 [file 40364_2025_793_MOESM1_ESM.docx]

**SUPPLEMENTARY MATERIAL**

**Supplementary Text**

1. **Cancer cohort**
2. **General inclusion and exclusion criteria**

The other inclusion criteria included measured creatinine clearance > 30 mL/min or creatinine ≤ 1.5 x ULN; aspartate aminotransferase (AST) and alanine aminotransferase (ALT) ≤ 2.5 x ULN (or ≤ 5 x ULN, in case of liver metastases); and serum bilirubin ≤ 1.5 x ULN. Specific biological criteria applied for the hepatocellular carcinoma cohort (Suppl Material).

Exclusion criteria for curative-intent cohorts comprised a history of previous cancers, except for adequately treated non-melanoma skin cancer, curatively treated in-situ cancer of the cervix with no evidence of disease for ≥ 5 years. The same criteria applied for non-curative-intent groups, except that patients with a recurrent cancer could have previously been treated with a curative intent treatment.

1. **Specific biological inclusion criteria applied for the hepatocellular carcinoma cohort**.

• Aspartate aminotransferase / AST and Alanine aminotransferase / ALT levels are accepted up to ≤ 5 x ULN

• Serum bilirubin ≤ 2.5 x ULN

• Absence or chronic hepatic encephalopathy, absence of refractory ascites, prothrombin rate ≥40% (or factor V ≥ 40% in case of antivitamin K therapy, albuminemia ≥ 25 g/L).

1. **Inclusion criteria for the curative intent cohort**. For curative-intent treatment groups, a prior anti-cancer treatment was allowed if this treatment was performed with curative intent, and if it did not include systemic chemotherapy, and if a complete remission ≥ 6 months was observed in between the end of treatment and relapse. Previous local treatments for superficial lesions were allowed without any time restriction (for example among others, intravesical treatment for superficial bladder cancer lesions). For non-curative treatment groups, patients were naïve of previous anti-cancer treatments considered as standard for metastatic disease.
2. **Amendments**: The first amendment adopted in 2019 allowed to modify blood drawing schedule at surgery or radiotherapy in order to fit with the patients visit diary; to add the post-operative kinetic cohort; to modify the ovarian cohort in order to include patients with platinum-sensitive relapse; to obtain the histological/cytological proof of cancer after inclusion, with the possibility to retrospectively exclude the patient in absence of pathological proof cancer. The 2020 amendment made some modifications on the cohorts initially planned: reduction in the number of patients planned in the gastric cohort (N=10); increase of patients to be enrolled in the head and neck cohort up to 50 patients; inclusion of a specific cohort of patients with NSCLC treated with immunotherapy (N=20); removing of the melanoma cohort (N=20); addition of a cohort of patients with thyroid cancer (N=10).

The 2021-2022 amendments also introduced minor changes in the protocol: participation of patients exclusively to the nychthemeral cohort, increase of the number of matched healthy controls and extension of the inclusion period.

In addition to hPG_80_, the following biomarkers were measured at the same times: AST; ALT; serum bilirubin (in addition to albumin, factor V et prothrombin time for HCC), serum creatinine, creatinine clearance, C-Reactive Protein (CRP); serum tumor markers for specific cohorts (CA15-3 and Carcinoembryonic antigen (CEA) for breast cancer, Prostate-specific antigen (PSA) for prostatic cancer, alpha foetoprotein (AFP) for HCC, CA19-9 and CEA for colorectal, gastric, superficial oesophago-gastric and pancreatic cancers, thyroglobulin for thyroid cancer, and CA125 for ovarian cancer and endometrial cancers).

1. **Asymptomatic healthy subjects control group**

The exclusion criteria included: any known personal history of cancers, or pre-cancerous lesions such as atypical dysplasia, in situ carcinomas; active smoking or previous smoking habit > 5 Pack-Year); a medical daily treatment (except for contraceptive, endocrine treatment for menopause, anti-hypertensive treatment, psychotropic treatment and anti-lipemic treatment provided that the inherent disease had been under control for at least 3 months).

**ELISA DxPG_80_.Lab kit (Biodena Care, Grabels, France)**

The limit of Detection (LoD) was set at a hPG_80_ concentration of 1.2 pM and the limit of Quantitation (LoQ) at a hPG_80_ concentration of 3.3 pM. The inter- and intra-assay coefficients of variation (CV%) were below 10%. No cross-reactivity was detected with gastrin-17, Gastrin-Gly or CTFP (C-Terminus Flanking Peptide). No cross-reactivity was detected with other blood biomarkers such as CA125, CEA or PSA. No interference was detected with chemicals and endogenous compounds such as SN-38, 5-FU or triglycerides, cholesterol or hemoglobin.

**Amendments**

Five main amendments were made between June 2018 and January 2023. Two of them introduced significant changes in the composition of cohorts to optimize the recruitment rate (e.g. suppression of a melanoma cohort replaced by a NSCLC cohort treated with immunotherapy; increase of the head-and-neck cancer cohort up to 50 patients; suppression of the “weekly cohort” meant to assess the hPG_80_ kinetics on a weekly basis during the first month of treatment) (Suppl Material).

As a consequence, the number of patients to enroll increased up to 420 (compared to the 410 initially planned).

**Supplementary Tables**

Suppl Table 1. Characteristics of patients with non-small lung cancers (NSCLC) in the non-curative intent cohort based on the major somatic & germline mutations, and tobacco status

| BASELINE CHARACTERISTICS | |
| --- | --- |
| **Lung cancer** | |
| ***Somatic mutations*** | ***N mutations (% of patients)*** |
| *BRAF V600* | *1 (2.22)* |
| *KRAS G12C* | *7 (15.56)* |
| *Other KRAS* | *7 (15.56)* |
| *EGFR* | *1 (2.22)* |
| *HER2* | *0 (0)* |
| *Others* | *6 (13.33)* |
| *Lack of mutations* | *11 (24.44)* |
| *Unknown/ not done* | *10 (22.22)* |
| ***Circulating tumor DNA mutations*** | ***N mutations (% of patients)*** |
| *TP53* | *14 (31.1)* |
| *EGFR* | *1 (2.2)* |
| *KRAS* | *9 (20.0)* |
| *BRAF* | *4 (8.9)* |
| ***Smoking status*** | ***N mutations (% of patients)*** |
| *Active smokers* | *20 (44.44)* |
| *0-20 pack years* | *3 (6.67)* |
| *21-40 pack years* | *7 (15.56)* |
| *> 40 pack years* | *8 (17.78)* |
| *Unknown* | *2 (4.44)* |
| *Former smokers* | *22 (48.89)* |
| *< 6 months* | *3 (6.67)* |
| *> 6 months* | *19 (42.22)* |
| *Unknown* | *0 (0)* |
| *Non-smokers* | *3 (6.67)* |

Suppl Table 2. Characteristics of hepatocellular carcinoma patients based on alcohol consumption, and cirrhosis

| **Hepatocellular carcinoma** | |
| --- | --- |
| ***Alcohol consumption*** | ***N (% of patients)*** |
| *Curative intent* | *40 (80.0)* |
| *Yes* | *10 (20.0)* |
| *No* | *30 (20.6)* |
| *Non-curative intent* | *10 (20.0)* |
| *Yes* | *2 (4.0)* |
| *No* | *8 (8.0)* |
| ***Cirrhosis*** | ***N (% of patients)*** |
| *Curative intent* | *40 (80.0)* |
| *Yes* | *29 (58.0)* |
| *No* | *11 (22.0)* |
| *Non-curative intent* | *10 (20.0)* |
| *Yes* | *5 (10.0)* |
| *No* | *5 (10.0)* |

**Supplementary Figures**

**
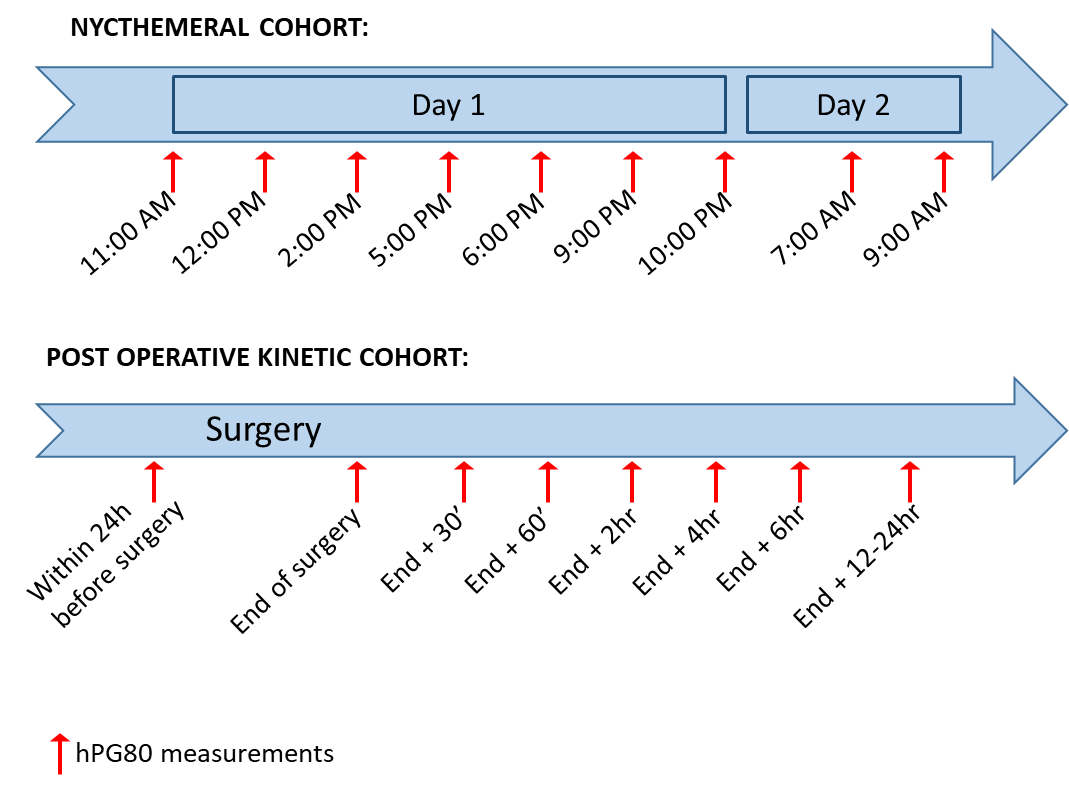
**

Suppl Figure 1. Sampling strategy in the nycthemeral and post-operative cohorts


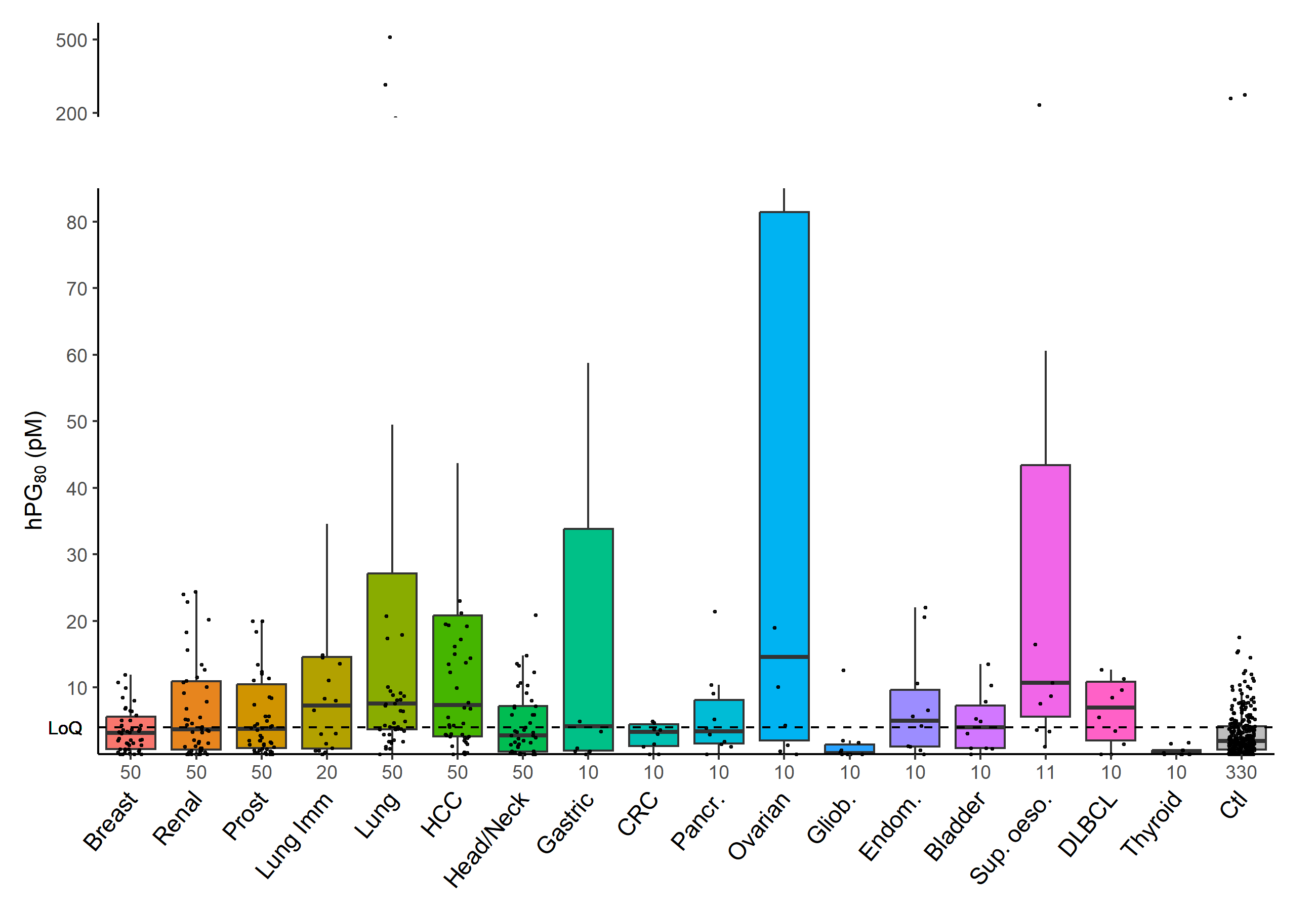


Suppl Figure 2. hPG_80_ concentrations in cancer patients from all cohorts and in the healthy subjects (Control: Ctl). The number of patients is indicated on the x-axis. Prost. Prostate; HCC: Hepatocellular carcinoma; Pancr.: Pancreas; Gliob.: Glioblastoma; Endom.: Endometrial; Sup. Oeso.: Superior Oesophagus; DLBCL: Diffuse Large B-Cell Lymphoma.


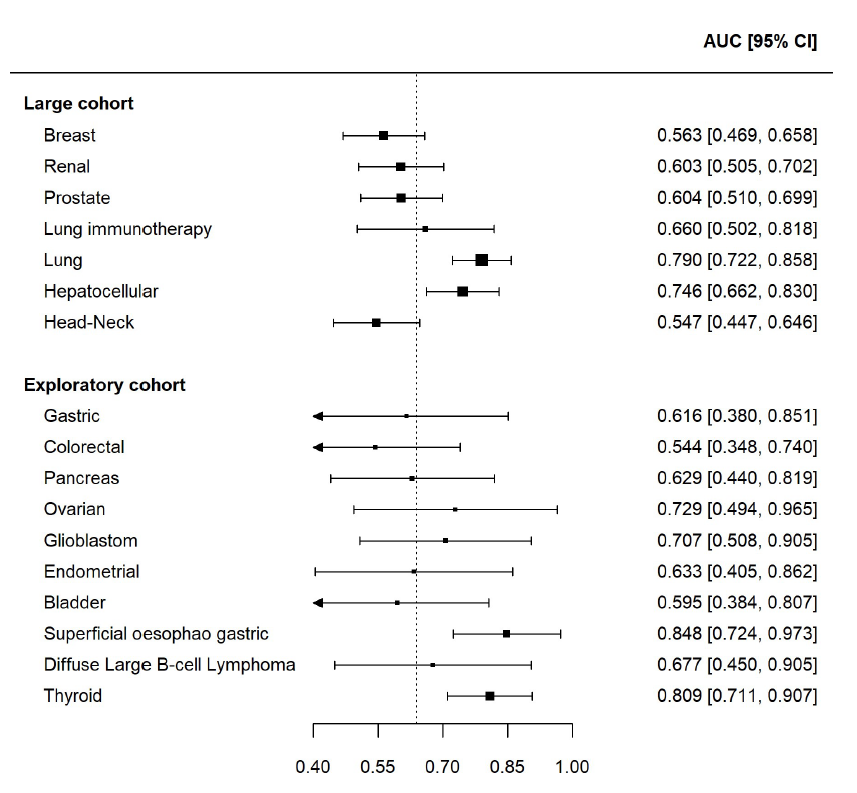


Suppl Figure 3. ROC are under the curve (AUC) by cohorts


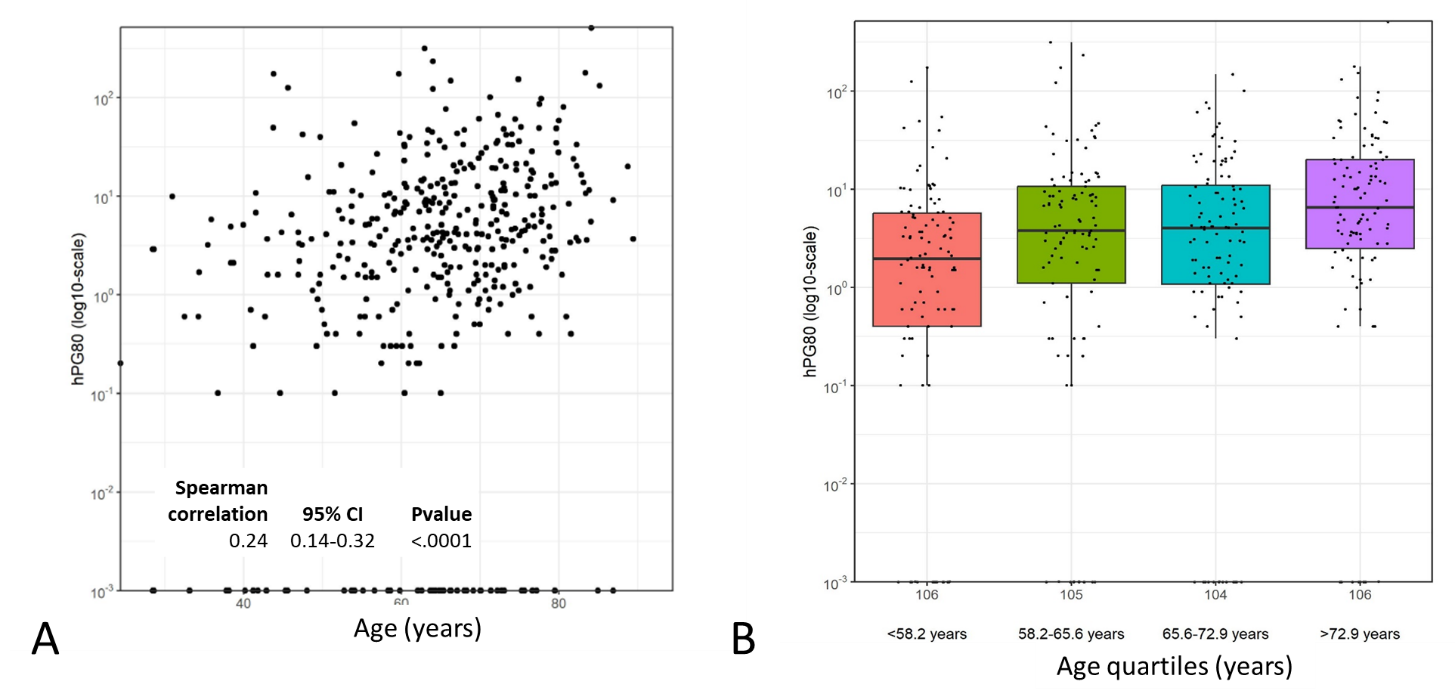


Suppl Figure 4. Relationships between baseline Log(hPG_80_) and patient age in the cancer cohort. (A) Scatter plot for all patients; (B) Box plots according to age quartiles.


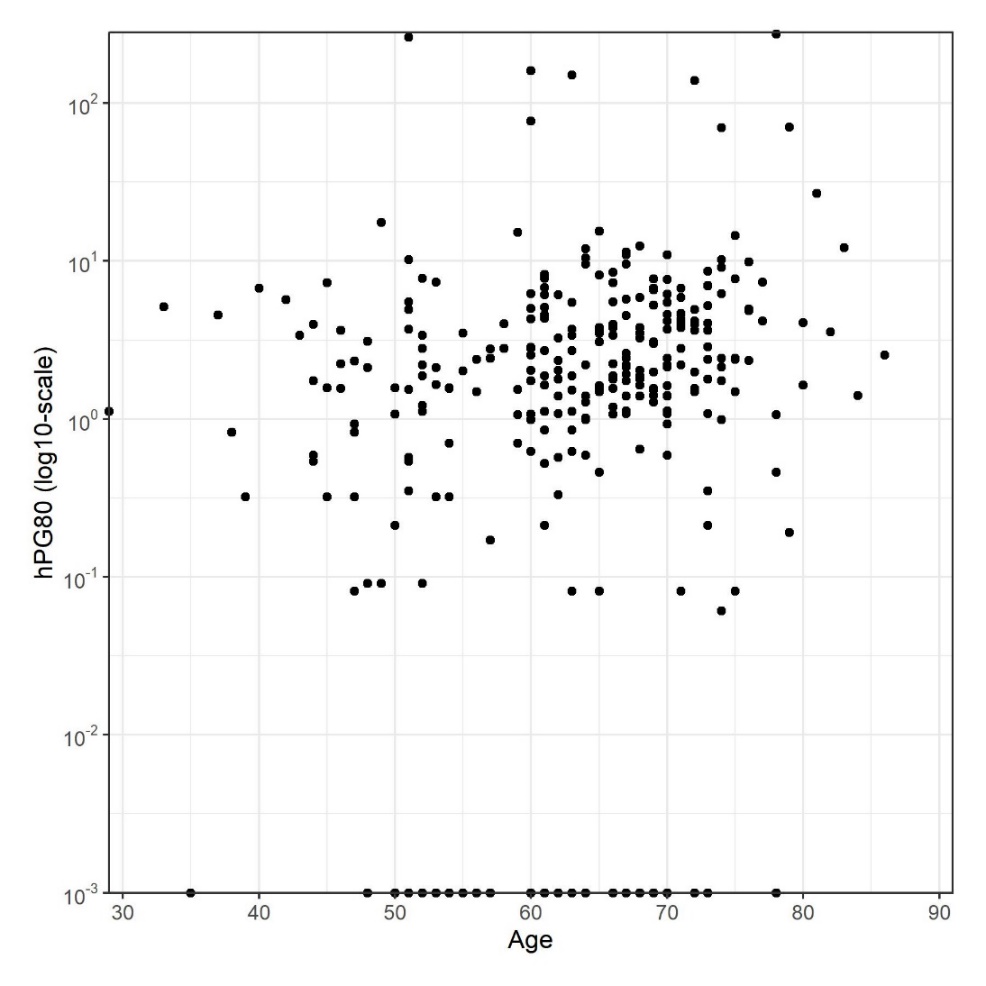


Suppl Figure 5. Relationships between baseline Log(hPG_80_) and age in the asymptomatic healthy subjects


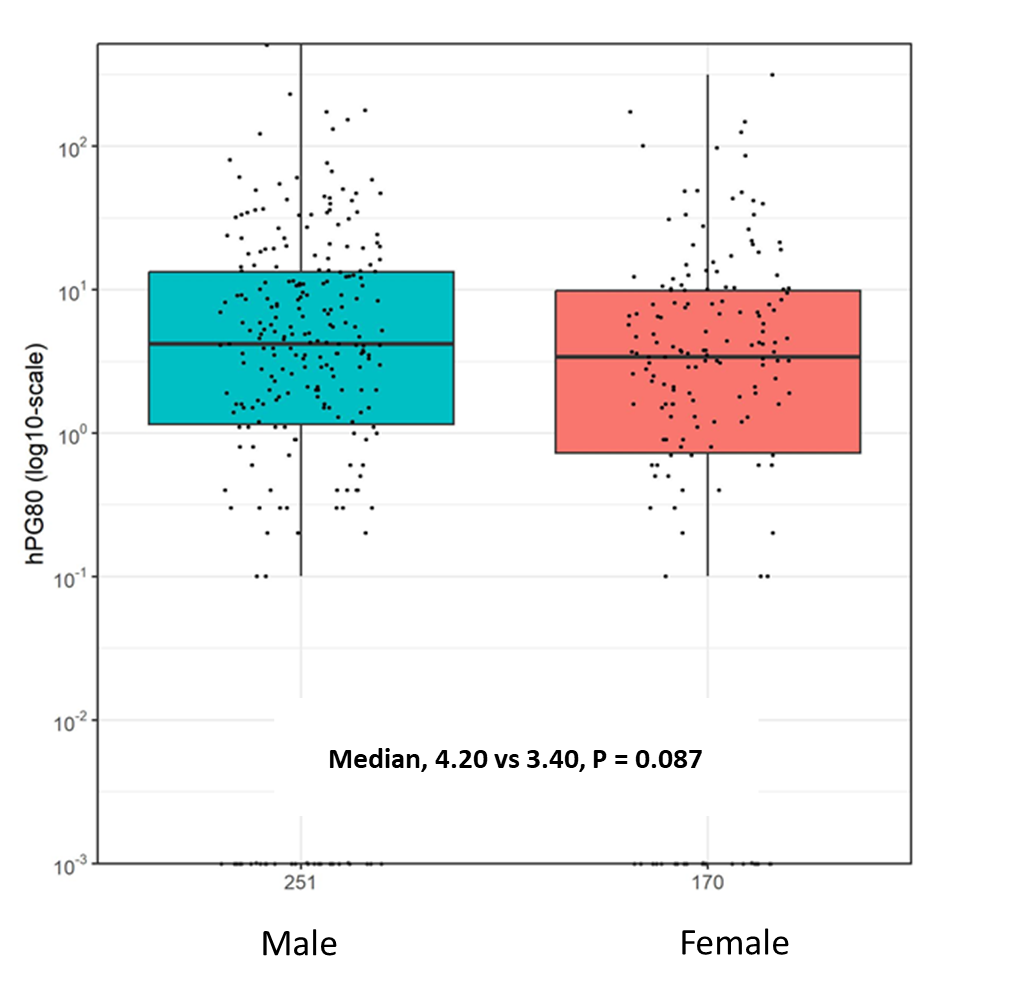


Suppl Figure 6. Relationships between baseline Log(hPG_80_) and gender in the cancer cohort


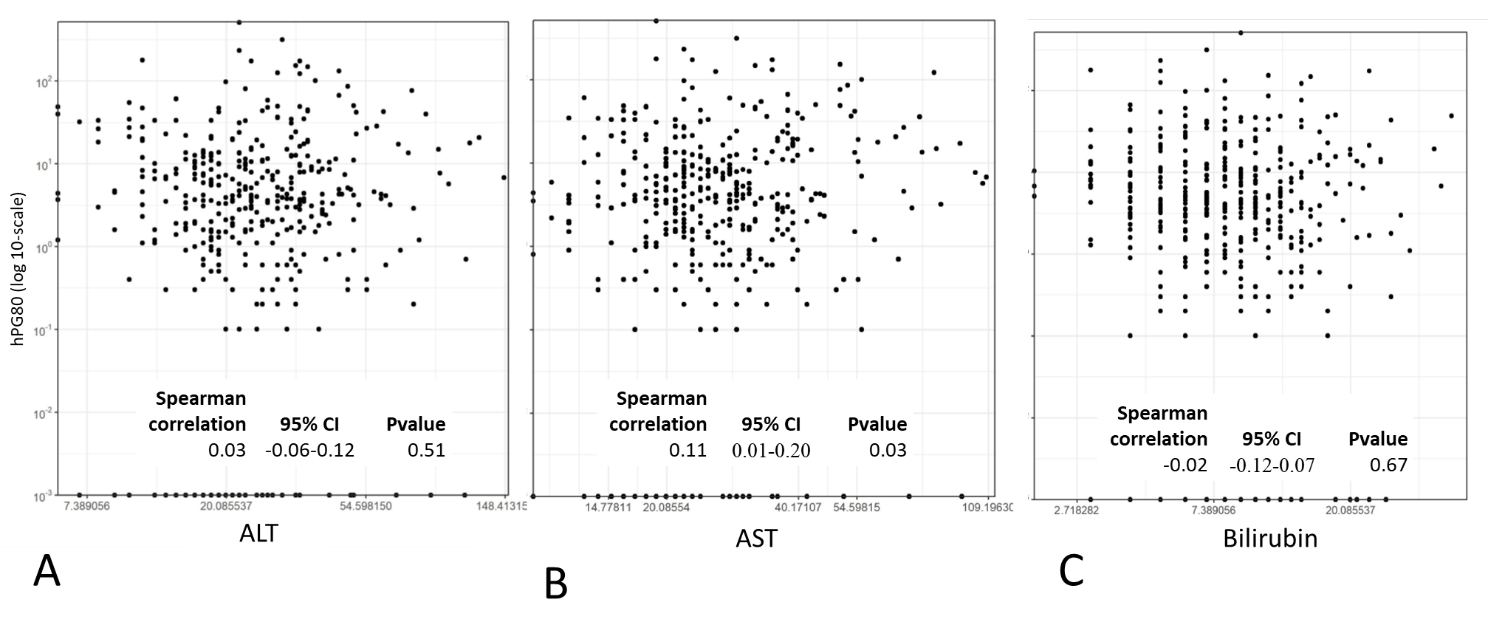


Suppl Figure 7. Correlations between baseline Log(hPG_80_) and liver function based on ALT (A); AST (B); and bilirubin (C).


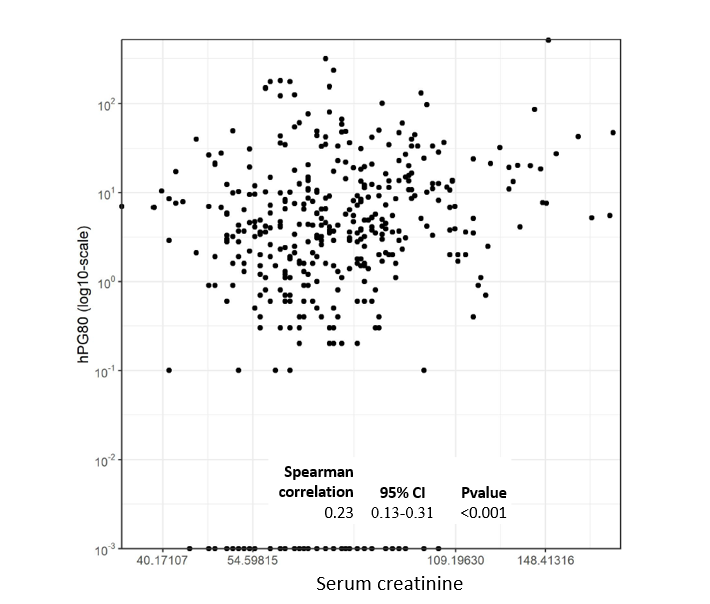


Suppl Figure 8. Correlation between baseline Log(hPG_80_) and serum creatinine.


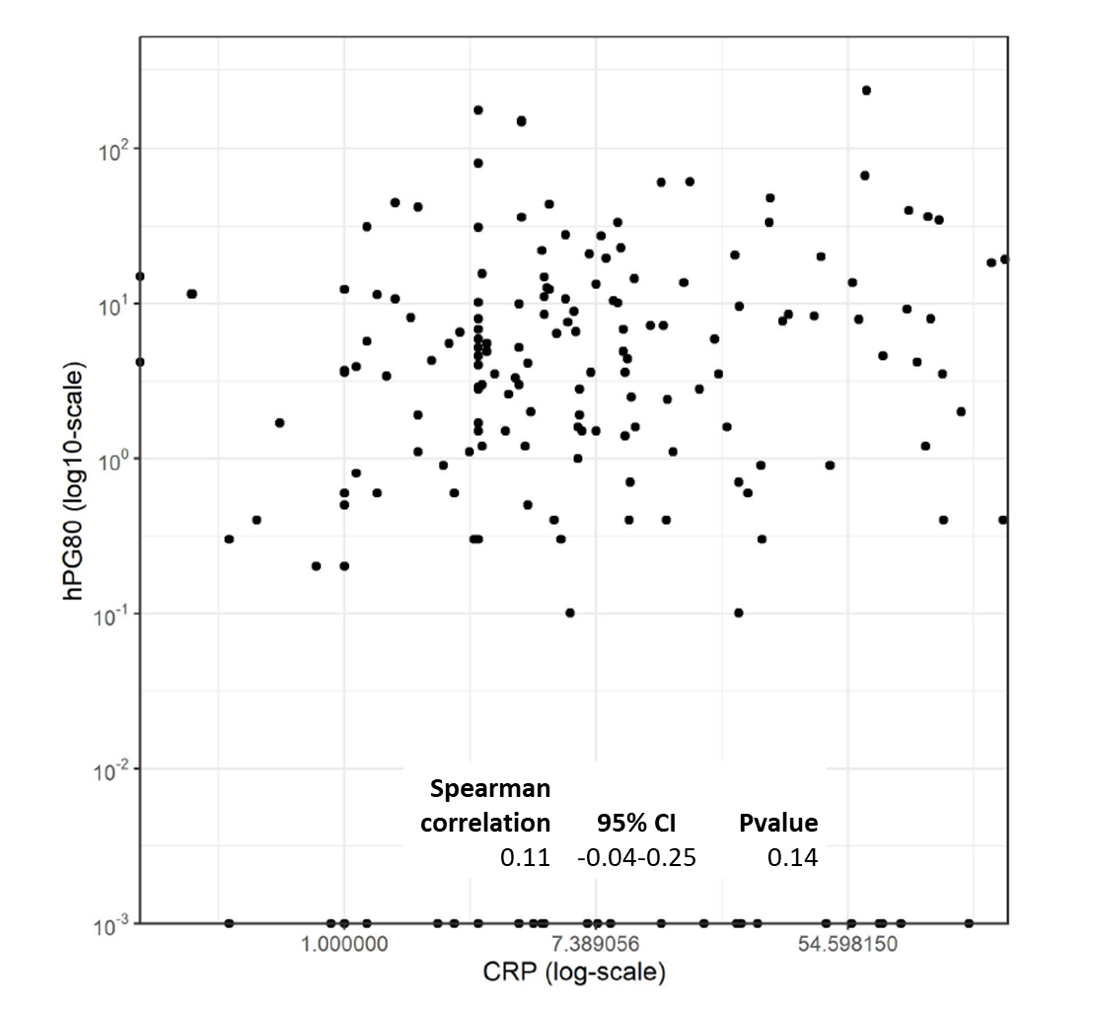


Suppl Figure 9. Correlation between baseline Log(hPG_80_) and C reactive protein.


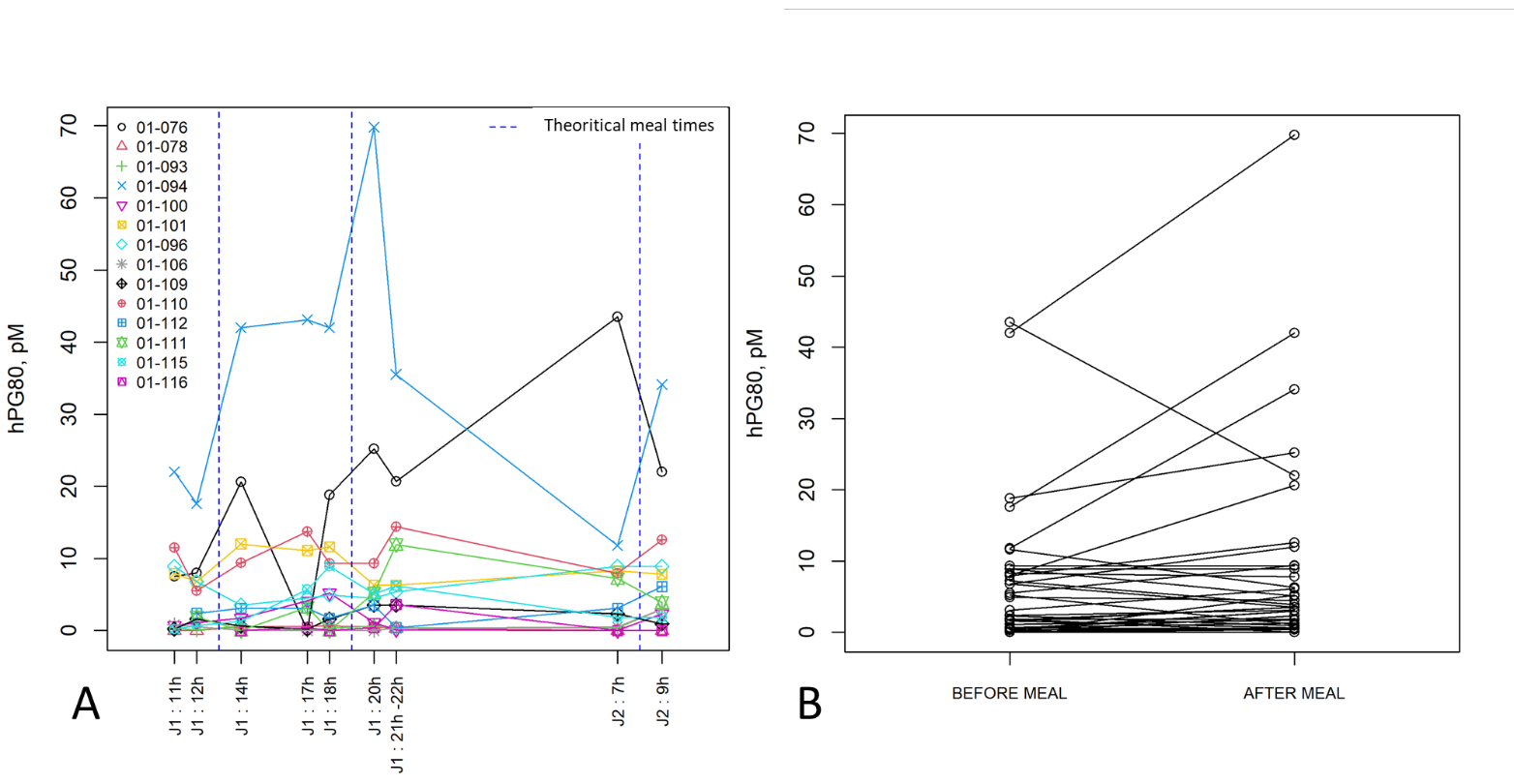


Suppl Figure 10. Kinetics of hPG_80_ versus time in the nychthemeral cohort, with (A) all time points and (B) before/after meal time points.


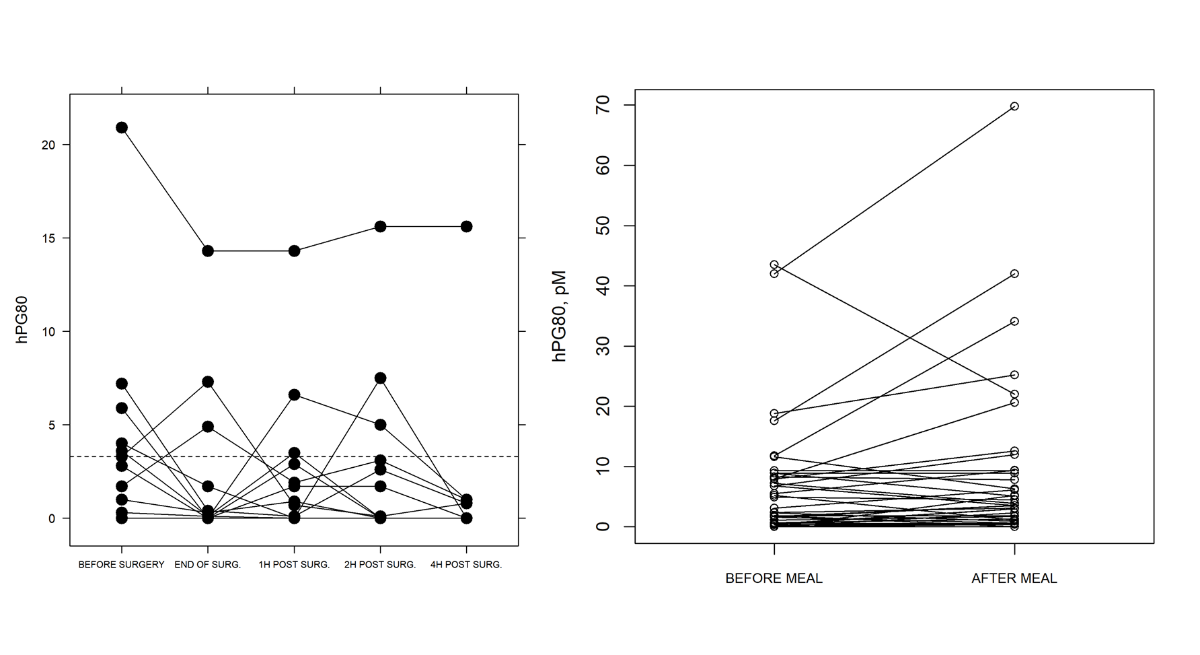


Suppl Figure 11. Kinetics of hPG_80_ versus time in the post-operative cohort.


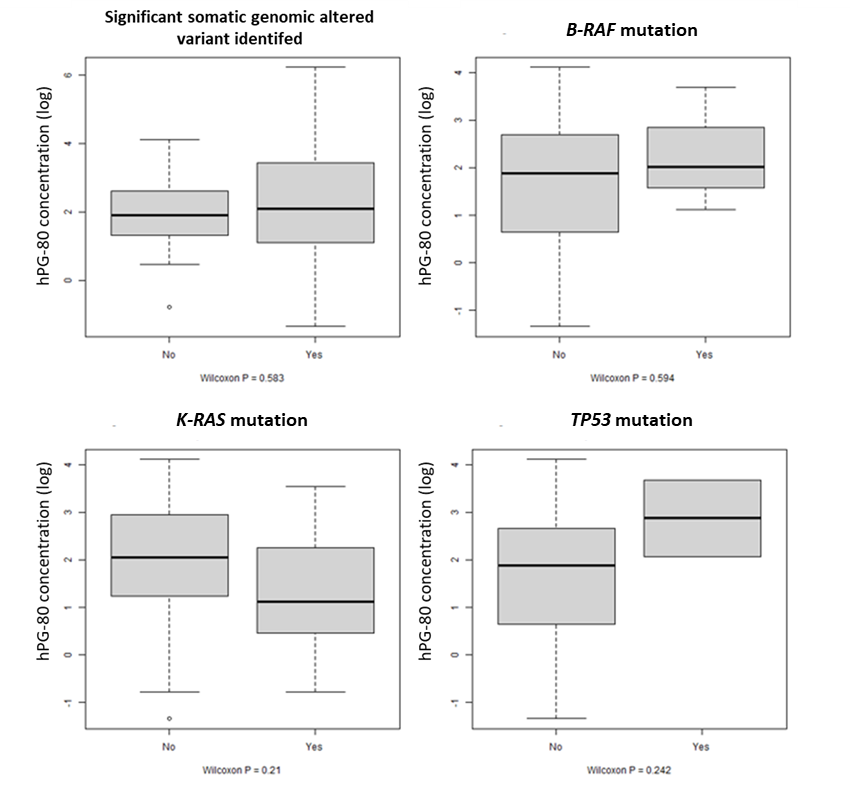


Suppl Figure 12. Baseline Log(hPG_80_) according to somatic mutations on tumor cells in the NSCLC cancer patients from the non-curative cohort


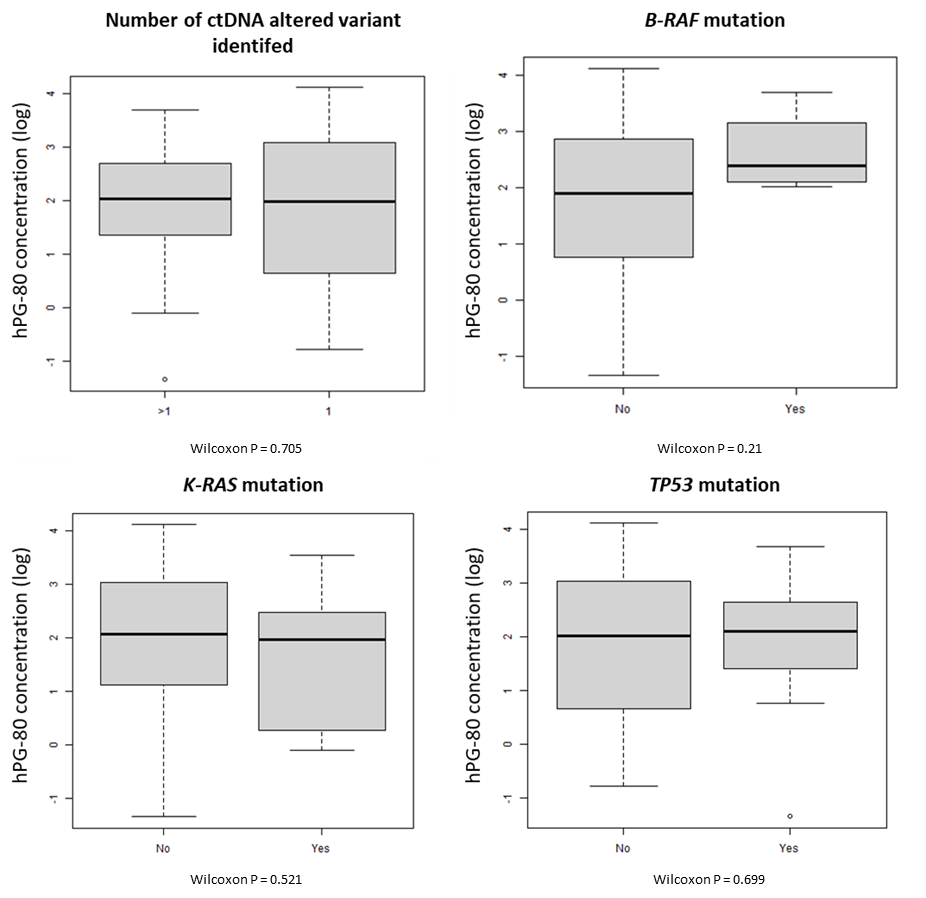


Suppl Figure 13. Baseline Log(hPG_80_) according to circulating tumor DNA (ctDNA) mutations in the NSCLC cancer patients from the non-curative cohort


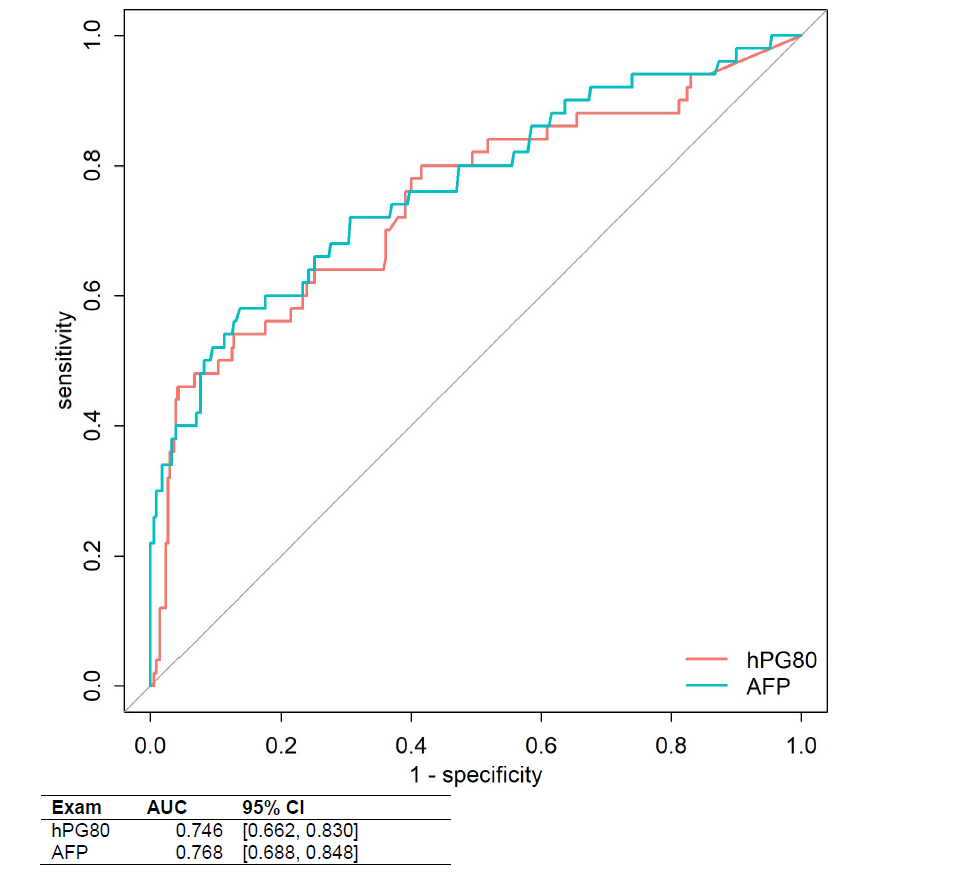


Suppl Figure 14. ROC curves of hPG_80_ and AFP in the hepatocellular carcinoma cohort


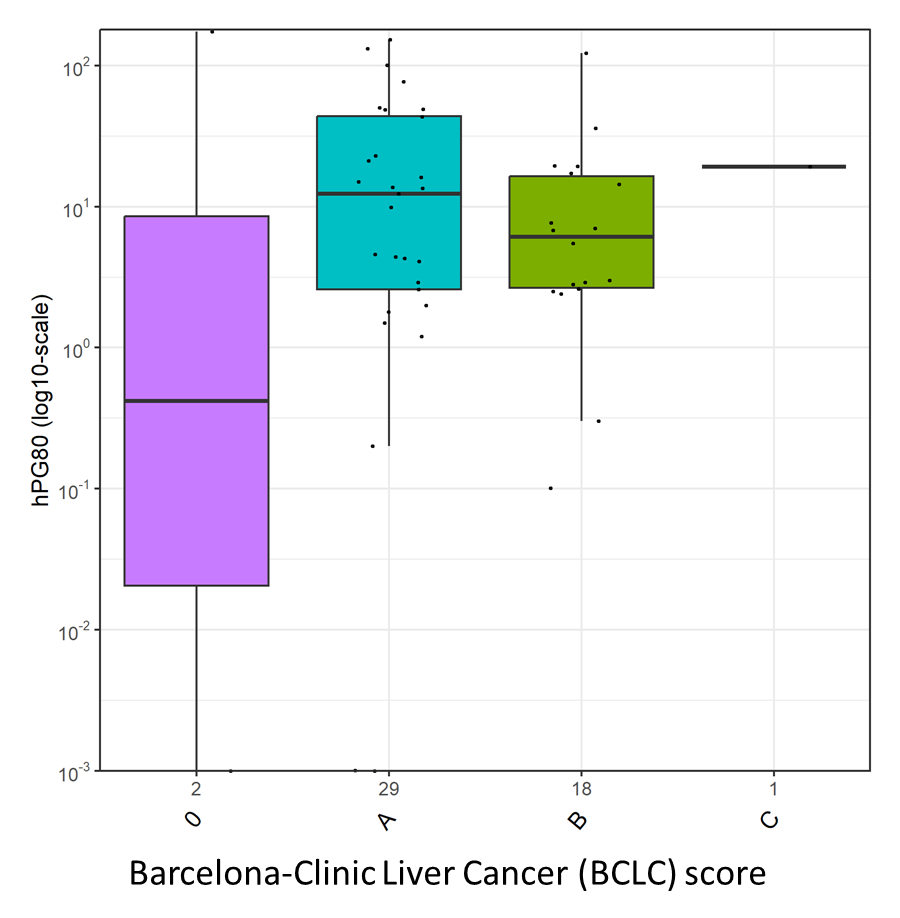


Suppl Figure 15. Baseline Log(hPG_80_) according to Barcelona-Clinic Liver Cancer (BCLC) score
